# Deep Learning Models to Screen Electronic Health Records for Breast and Colorectal Cancer Progression: Performance Evaluation Study

**DOI:** 10.2196/63767

**Published:** 2025-10-13

**Authors:** Pascal Lambert, Rayyan Khan, Marshall Pitz, Harminder Singh, Helen Chen, Kathleen Decker

**Affiliations:** 1 Paul Albrechtsen Research Institute CancerCare Manitoba Winnipeg, MB Canada; 2 Department of Epidemiology and Cancer Registry CancerCare Manitoba Winnipeg, MB Canada; 3 Department of Electrical and Computer Engineering University of Manitoba Winnipeg, MB Canada; 4 Department of Internal Medicine University of Manitoba Winnipeg, MB Canada; 5 Department of Medical Oncology and Hematology CancerCare Manitoba Winnipeg, MB Canada; 6 Department of Community Health Sciences University of Manitoba Winnipeg, MB Canada; 7 School of Public Health Science University of Waterloo Waterloo, ON Canada

**Keywords:** breast cancer, colorectal cancer, cancer progression, natural language processing, retrospective chart review, Bio+ClinicalBERT, Clinical-BigBird, Clinical-Longformer

## Abstract

**Background:**

Cancer progression is an important outcome in cancer research. However, it is frequently documented only in electronic health records (EHRs) as unstructured text, which requires lengthy and costly chart reviews to extract for retrospective studies.

**Objective:**

This study aimed to evaluate the performance of 3 deep learning language models in determining breast and colorectal cancer progression in EHRs.

**Methods:**

EHRs for individuals diagnosed with stage 4 breast or colorectal cancer between 2004 and 2020 in Manitoba, Canada, were extracted. A chart review was conducted to identify cancer progression in each EHR. Data were analyzed with pretrained deep learning language models (Bio+ClinicalBERT, Clinical-BigBird, and Clinical-Longformer). Sensitivity, positive predictive value, area under the curve, and scaled Brier scores were used to evaluate performance. Influential tokens were identified by removing and adding tokens to EHRs and examining changes in predicted probabilities.

**Results:**

Clinical-BigBird and Clinical-Longformer models for breast and colorectal cancer cohorts demonstrated higher accuracy than the Bio+ClinicalBERT models (scaled Brier scores for breast cancer models: 0.70-0.79 vs 0.49-0.71; scaled Brier scores for colorectal cancer models: 0.61-0.65 vs 0.49-0.61). The same models also demonstrated higher sensitivity (breast cancer models: 86.6%-94.3% vs 76.6%-87.1%; colorectal cancer models: 73.1%-78.9% vs 62.8%-77.0%) and positive predictive value (breast cancer models: 77.9%-92.3% vs 80.6%-85.5%; colorectal cancer models: 81.6%-86.3% vs 72.9%-82.9%) compared to Bio+ClinicalBERT models. All models could remove more than 84% of charts from the chart review process. The most influential token was the word *progression*, which was influenced by the presence of other tokens and its position within an EHR.

**Conclusions:**

The deep learning language models could help identify breast and colorectal cancer progression in EHRs and remove most charts from the chart review process. A limited number of tokens may influence model predictions. Improvements in model performance could be obtained by increasing the training dataset size and analyzing EHRs at the sentence level rather than at the EHR level.

## Introduction

### Background

Cancer progression is an important intermediate prognostic and predictive marker of clinical outcomes among patients with cancer. It is defined as the growth or spread of an existing cancer. Progression is distinct from cancer recurrence, which is the diagnosis of a second episode of cancer after the first episode is considered cured. Unfortunately, cancer progression and its date of occurrence are frequently not available in cancer registries and are only documented in the electronic health record (EHR) as unstructured text. Reports such as pathology and radiology reports also contain text that can inform the diagnosis of cancer progression in clinical notes. Therefore, the inclusion of cancer progression in retrospective research requires a chart review, which can be lengthy and expensive. Consequently, researchers may not have been able to include progression as an outcome in retrospective studies.

Algorithms using administrative claims data (eg, diagnosis and procedure codes and pharmacy-dispensed medication) have been developed to identify cancer recurrence and progression [1-3]. However, these algorithms do not approximate case definitions obtained from chart reviews well. When algorithms demonstrated sensitivity values >90%, the positive predictive values (PPV) were only near or <50%. PPV can be low due to algorithms misclassifying changes in treatment as cancer progression or recurrence when the change was due to other reasons (eg, toxicity). In addition, most algorithms demonstrated sensitivity values <100%. Methods other than algorithms that use administrative claims data are needed to reduce misclassifications and improve accuracy.

One recent option is to use pretrained deep learning language models, which have the potential to improve model accuracy relative to claims-based algorithms while decreasing the need for lengthy and costly chart reviews. Bidirectional encoder representations from transformers (BERT) [4] is an example of such a model, which can be fine-tuned to datasets to perform tasks such as creating classification models. This approach has demonstrated higher accuracy in developing text classification models than previous deep learning language models (eg, long short-term memory models [4]). Rather than using proxy information for identifying cancer progression (eg, claims data), these language models can analyze data directly from EHRs.

### Objectives

The objective of this study was to develop and compare models to identify cancer progression in EHRs for stage 4 breast and colorectal cancer cohorts using 3 pretrained deep learning language models.

## Methods

### Data

Stage 4 breast and colorectal cancer cohorts were identified from the Manitoba Cancer Registry. Individuals diagnosed between January 1, 2004, and December 31, 2020, were included. EHRs for physician visits on or after an individual’s diagnosis date were extracted from the CancerCare Manitoba’s ARIA system up to April 30, 2022. Only text from physician notes within EHRs was extracted because these records included notes such as physicals and histories, progression notes, and treatment plans that would discuss the suspicion, investigation, and diagnosis of progression. Notes from other professionals (eg, nutritionists, social workers, and nurses) were removed because the diagnosis of cancer progression is the responsibility of physicians. Radiology and pathology reports as source documents were excluded. Radiology and pathology reports were stored as PDF files, which had issues with extracting text and retaining proper format (eg, text from multiple columns would be read as a single line rather than separate lines from different columns). Training and validation breast cancer cohorts included EHRs from individuals diagnosed between January 1, 2004, and December 31, 2017. Training and validation colorectal cancer cohorts included EHRs from individuals diagnosed between January 1, 2011, and December 31, 2017. Training and validation cohorts consisted of weight-stratified datasets representing 80% and 20% of the EHRs, respectively. Test cohorts consisted of EHRs from individuals with breast or colorectal cancer diagnosed between January 1, 2018, and December 31, 2020. Temporal external validation was used because external validation is more rigorous than internal validation [5].

Cancer progression reported in clinical notes was defined as a requirement for treatment change due to tumor growth. Tumor growth could be determined radiologically, when new lesions appeared or had increased in size on imaging. Criteria such as the response evaluation criteria in solid tumors may or may not have been reported in clinical notes. Tumor growth could also be considered by rising blood marker values or pathologically (eg, new lesions). Progression could also be defined clinically when deterioration requires a change in treatment, such as enrollment in palliative care.

Labels were provided for each EHR to identify cancer progression. Three-class labels were considered: (1) cancer progression (reported by an oncologist in the clinical note using the previously mentioned factors); (2) mention of progression but not actual progression or suspicion of progression (which occurred for 1 of 3 reasons—tumor growth was reported in the clinical note as not having increased enough to justify a change in treatment; nononcological use, eg, “the patient’s symptoms are progressing well”; or suspicion of progression without sufficient evidence to indicate a diagnosis); and (3) no mention of progression. Three-class labels were generated to potentially reduce false-positive rates. Two trained research assistants reviewed EHRs for the breast cancer cohort, 2 other trained research assistants reviewed EHRs for the colorectal cancer cohort, and a third individual with prior training provided the final label when disagreements in labels occurred between chart reviewers.

### Analysis

Preprocessing of data before analysis consisted of changing all text to lowercase and removing words that contained more than 50 characters. Some EHR notes had *PROGRESS NOTE* as the first header. This text was removed to prevent models from confusing this term with cancer progression. Three pretrained deep learning language models (Bio+ClinicalBERT [6], Clinical-BigBird [7], and Clinical-Longformer [7]) were used to analyze data. Bio+ClinicalBERT is limited to a maximum of 512 tokens, whereas Clinical-BigBird and Clinical-Longformer are limited to a maximum of 4096 tokens. Three approaches were used with Bio+ClinicalBERT to account for the 512-token maximum: analyzing the first 512 tokens (head), analyzing the last 512 tokens (tail), and analyzing the first 256 and last 256 tokens (head and tail). Five-fold cross-validation was used with a repeated weighted k-fold function. The following parameters were used to optimize the results on the validation dataset: batch size (16 and 32 for Bio+ClinicalBERT; 4 for Clinical-BigBird and Clinical-Longformer), learning rate (1e-2 and 1e-5), and weight decay (0 and 0.25). Up to 10 epochs were considered with a stopping rule (a patience of 3 for Bio+ClinicalBERT and a patience of 1 for Clinical-BigBird and Clinical-Longformer). When the parameters were determined, the analysis was completed by combining the training and validation cohorts. The developed models were then applied to the test datasets. The models were analyzed with 2-class labels (cancer progression vs other labels combined) and 3-class labels. The models were run using a workstation with 2 GPUs of 24 GB RAM each.

### Model Performance

Accuracy (ie, correct classification) was used to compare model performance by epoch. After the epoch numbers were selected, the model performance was evaluated on the validation and test cohorts. Performance measures for evaluating the 3-class models were based on the predicted probabilities of the “cancer progression” category versus the other 2 categories combined. Reported measures included sensitivity, PPV, area under the curve (AUC; a measure of discrimination), scaled Brier score (a measure of predictive accuracy), and the percentage of charts reduced (ie, true negatives plus false negatives divided by the total number of EHRs). The probability threshold used for sensitivity and PPV was 0.50. Models generated with breast cancer data were applied to the colorectal cancer test dataset, and vice versa, to evaluate generalizability.

Additional output was generated when evaluating the test datasets. The rate of progression over time was calculated for the observed data using the Kaplan-Meier (KM) approach. The predicted rate of progression over time was generated by assuming that the models would be used as a screening tool to reduce the number of charts that need to be reviewed or to replace the need for a chart review. When evaluating the model as a screening tool to reduce the number of charts required to review, the time to first progression predicted by the model on or after the first observed EHR identifying cancer progression was used to calculate time to progression. This was done because when used as a screening tool, users should not identify cancer progression before the observed date. In contrast, when evaluating the model to replace the need for a chart review, the time to the first EHR predicted by the model to mention progression was used to indicate time to progression. The hazard ratio (HR) from a Cox regression model with age at diagnosis (≥65 years vs <65 years) as a predictor was also run using the observed data. Similar to the KM analysis, Cox regression models were run, assuming that the models were used as a screening tool or to replace a chart review. Predicted values close to both observed KM rates and HRs indicate that a model approximated the observed data well.

### Model Interpretability

The detection of influential tokens was determined by selecting samples of EHRs identified as true positive, true negative, false positive, and false negative from the test cohorts. EHRs with predicted probabilities near 0.00, 0.50, and 1.00 were sampled. EHRs were modified by replacing individual words or partial sentences with nonsensical terms (eg, XXXXXX) or by adding words or partial sentences that were assumed to be influential. Common terms that describe cancer progression (eg, progression) were added or removed from EHRs. Partial sentences such as *no evidence of progression* and *has not progressed yet* were also added. The predicted probabilities of the original and modified EHRs were compared. If an EHR had a predicted probability near 1.00, individual words and partial sentences were removed until the predicted probability was decreased to near 0.

### Ethical Considerations

The study was conducted according to the guidelines of the Declaration of Helsinki and was approved by the University of Manitoba’s Health Research Ethics Board (HS21379 [HS2017:422]; approval date March 28, 2022), the University of Waterloo’s Ethics Board (44261; approval date April 29, 2022), the Manitoba Health’s Provincial Health Research Privacy Committee, and the CancerCare Manitoba’s Research and Resource Impact Committee. Because data were retrospectively collected and analyzed, informed consent was not required, and compensation was not provided. No identifiable information was included in the manuscript to further maintain and ensure privacy and confidentiality.

## Results

### Breast Cancer

In the breast cancer training (19,031 EHRs) and validation (4758 EHRs) datasets, 3884 (16.33%) EHRs indicated cancer progression, and 4324 (18.18%) indicated progression mentioned but not present (ie, ratios of 0.24 and 0.27 relative to the *no cancer progression* class). In the breast cancer test dataset (4486 EHRs), 582 (12.97%) EHRs indicated cancer progression, and 817 (18.21%) indicated progression mentioned but not present (ie, ratios of 0.19 and 0.26 relative to the *no cancer progression* class). The number of tokens per chart differed by the model tokenizer (Table 1). All 3 tokenizers reported that more than 30% (breast cancer training and validation data: 8860/23,789, 7719/23,789, and 7775/23,789; breast cancer test data: 1955/4758, 1724/4758, and 1737/4758) of EHRs had more than 512 tokens and that no EHRs had more than 4096 tokens.

**Table 1:** Number of tokens by tokenizers.

Model and metric		Breast cancer		Colorectal cancer	
		Training and validation (n=23,789)	Test (n=4486)	Training and validation (n=23,977)	Test (n=6045)
**Bio+ClinicalBERT**					
	Mean (SD)	486 (323)	548 (358)	488 (316)	547 (357)
	Maximum	2821	2717	2987	3619
	EHRs^a^ >512, n (%)	8860 (37.24)	1955 (43.58)	9186 (38.31)	2744 (42.84)
**Clinical-BigBird**					
	Mean (SD)	451 (300)	506 (331)	451 (292)	505 (331)
	Maximum	2569	2523	2784	3342
	EHRs >512, n (%)	7719 (32.45)	1724 (38.43)	7866 (32.81)	2368 (36.97)
**Clinical-Longformer**					
	Mean	453 (301)	508 (332)	454 (293)	508 (333)
	Maximum	2584	2528	2796	3353
	EHRs >512, n (%)	7775 (32.68)	1737 (38.72)	7966 (33.22)	2401 (37.49)

^a^EHR: electronic health record.

Only 1 epoch was required for the breast cancer models. Bio+ClinicalBERT reached 99% to 100% accuracy in the training dataset by the fourth or fifth epoch, but accuracy for the validation dataset was highest at the first epoch. Clinical-BigBird and Clinical-Longformer reached 99% to 100% accuracy in the training dataset by the second epoch, but accuracy for the validation dataset was highest at the first epoch.

The larger token models (Clinical-BigBird and Clinical-Longformer) demonstrated higher predictive accuracy (ie, scaled Brier scores) than the Bio+ClinicalBERT models in both validation and test datasets (Table 2). The highest sensitivity, PPV, and AUC were also achieved by the larger token models in the test datasets.

**Table 2:** Results by model and outcome class for breast cancer validation and test datasets.

Datasets and metrics		Bio+ClinicalBERT									Clinical-BigBird					Clinical-Longformer		
		2-class				3-class				2-class			3-class		2-class			3-class
		Head	Tail	Head and tail	Head		Tail	Head and tail										
**Validation**																		
	Sensitivity, %	71.9	71.2	80.7	79.0		84.2	74.0	84.0			86.0		76.7			*86.7* ^a^	
	PPV^b^, %	85.1	90.5	86.8	88.1		84.7	87.5	88.7			84.2		*93.0*			84.1	
	AUC^c^	0.96	0.97	0.97	0.97		*0.98*	0.97	*0.98*			*0.98*		0.97			*0.98*	
	Scaled Brier score	0.60	0.62	0.68	0.68		0.70	0.64	*0.73*			0.71		0.72			0.72	
	Charts reduced, %	86.2	*87.2*	84.8	85.4		83.8	86.2	84.5			83.3		86.5			83.2	
**Test**																		
	Sensitivity, %	84.5	76.6	87.1	82.3		85.9	82.5	86.6			93.6		*94.3*			93.6	
	PPV, %	85.1	87.1	81.8	85.5		80.6	83.6	*92.3*			77.9		78.7			81.6	
	AUC	0.98	0.97	0.98	0.98		0.98	0.98	*0.99*			*0.99*		*0.99*			*0.99*	
	Scaled Brier score	0.71	0.66	0.69	0.70		0.68	0.68	*0.79*			0.72		0.70			0.76	
	Charts reduced, %	87.1	*88.6*	86.2	87.5		86.2	87.2	87.8			84.4		84.4			85.1	

^a^Italicization indicates the highest value.

^b^PPV: positive predictive value.

^c^AUC: area under the curve.

**Figure 1:**
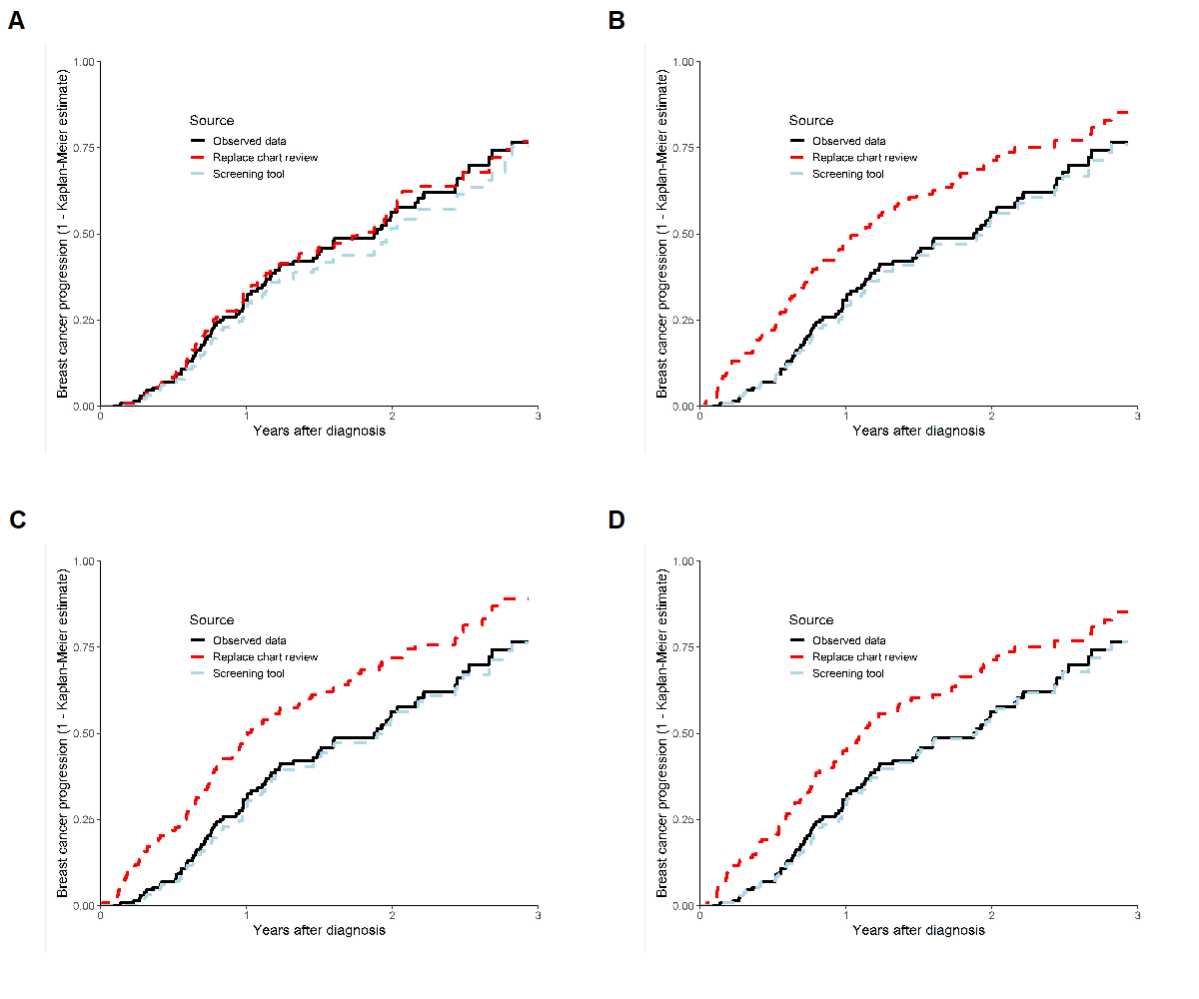
Kaplan-Meier curves of time to progression for the observed data and through the use of Clinical-BigBird and Clinical-Longformer in the breast cancer test dataset (A: 2-class Clinical-BigBird; B: 3-class Clinical-BigBird; C: 2-class Clinical-Longformer; and D: 3-class Clinical-Longformer).

The test dataset included 140 individuals, of whom 77 (55%) had at least 1 EHR indicating cancer progression. Because the larger token models indicated higher accuracy than the Bio+ClinicalBERT models, an additional output was generated to evaluate their performance. Model predictions were used to identify the first EHR after diagnosis that predicted cancer progression (to represent a model replacing a chart review) and the first EHR on or after the first EHR identifying cancer progression (to represent a model being used as a screening tool). The resulting data were then analyzed using KM curves. All 4 larger token models (Clinical-BigBird and Clinical-Longformer, each with 2- and 3-class outcomes) demonstrated good performance as a screening tool (Figure 1), where KM estimates derived were good approximations of observed cancer progression rates. In total, 3 of the 4 models demonstrated an overestimation of cancer progression when used to replace a chart review. The Clinical-BigBird 2-class model reported the highest PPV (92.3%) among the 4 models and demonstrated estimates very close to the observed data when used to replace a chart review. Similarly, when the data generated were included in Cox regression models with age at diagnosis as a predictor, all larger token models provided results similar to the observed data when used as a screening tool, and all larger token models, except the Clinical-Longformer 3-class model, provided results similar to the observed data when used to replace a chart review (Table 3).

**Table 3:** Cox regression estimates (age predictor: ≥65 years vs <65 years) for the breast cancer test cohort.

Model			HR^a^ (95% CI)
Observed			0.62 (0.39-0.98)
**Screening tool**			
	Clinical-BigBird 2-class	0.62 (0.39-1.01)	
	Clinical-BigBird 3-class	0.60 (0.37-0.96)	
	Clinical-Longformer 2-class	0.60 (0.37-0.95)	
	Clinical-Longformer 3-class	0.61 (0.38-0.97)	
**Replace chart review**			
	Clinical-BigBird 2-class	0.64 (0.40-1.01)	
	Clinical-BigBird 3-class	0.67 (0.45-1.00)	
	Clinical-Longformer 2-class	0.64 (0.43-0.96)	
	Clinical-Longformer 3-class	0.79 (0.52-1.18)	

^a^HR: hazard ratio.

### Colorectal Cancer

In the colorectal cancer training (19,182 EHRs) and validation (4795 EHRs) datasets, 2980 (12.42%) EHRs indicated cancer progression, and 3035 (12.66%) indicated progression mentioned but not present (ie, ratios of 0.16 and 0.17 relative to the *no cancer progression* class). In the colorectal test dataset (6405 EHRs), 683 (10.66%) EHRs indicated cancer progression and 807 (12.60%) indicated progression mentioned but not present (ie, ratios of 0.14 and 0.17 relative to the *no cancer progression* class). The number of tokens per chart differed by model tokenizer (Table 1). All 3 tokenizers reported that more than 30% (colorectal cancer training and validation data: 9186/23,977, 7866/23,977, and 7966/23,977; colorectal cancer test data: 2744/4975, 2368/4975, and 2401/4975) of EHRs had more than 512 tokens and that no EHRs had more than 4096 tokens.

Only 1 epoch was required for the colorectal cancer models. Bio+ClinicalBERT reached 99% to 100% accuracy in the training dataset by the fourth or fifth epoch, but accuracy for the validation dataset was highest at the first epoch. Clinical-BigBird and Clinical-Longformer reached 99% to 100% accuracy in the training dataset by the second epoch, but accuracy for the validation dataset was highest at the first epoch.

The Clinical-Longformer models reported the highest sensitivity and PPV for the validation dataset (Table 4). The Clinical-BigBird 3-class model reported the highest AUC and scaled Brier score for the validation dataset. The Bio+ClinicalBERT 2-class head and tail model reported the highest PPV and percentage of charts reduced. The highest sensitivity, PPV, AUC, and scaled Brier score for the test dataset were found with the larger token models, whereas the highest percentage of charts reduced was found with the Bio+ClinicalBERT 3-class tail model.

**Table 4:** Results by model and outcome class for colorectal cancer validation and test datasets.

Datasets and metrics		Bio+ClinicalBERT									Clinical-BigBird					Clinical-Longformer		
		2-class				3-class				2-class			3-class		3-class			3-class
		Head	Tail	Head and tail	Head		Tail	Head and tail										
**Validation**																		
	Sensitivity, %	80.9	78.5	82.0	86.4		79.9	80.4	82.4			88.4		81.0			*91.3* ^a^	
	PPV^b^, %	88.3	85.2	88.7	84.0		80.3	87.1	88.3			87.4		*91.8*			82.4	
	AUC^c^	0.98	0.98	0.97	*0.99*		0.98	0.98	0.98			*0.99*		0.98			*0.99*	
	Scaled Brier score	0.72	0.67	0.73	0.73		0.66	0.72	0.75			*0.77*		0.76			0.75	
	Charts reduced, %	88.6	88.6	88.5	87.2		87.6	88.5	88.4			87.4		*89*			86.2	
**Test**																		
	Sensitivity, %	74.1	62.8	71.9	77.0		63.7	69.7	78.5			75.4		73.1			*78.9*	
	PPV, %	79.8	81.1	82.4	72.9		82.4	82.9	81.6			82.0		*86.3*			81.9	
	AUC	0.96	0.95	0.96	0.96		0.95	0.96	*0.97*			0.96		0.96			*0.97*	
	Scaled Brier score	0.56	0.49	0.54	0.61		0.54	0.58	0.63			0.61		*0.65*			0.64	
	Charts reduced, %	90.1	91.7	90.7	88.7		*91.8*	91.0	89.7			90.2		91.0			89.7	

^a^Italicization indicates the highest value.

^b^PPV: positive predictive value.

^c^AUC: area under the curve.

**Figure 2:**
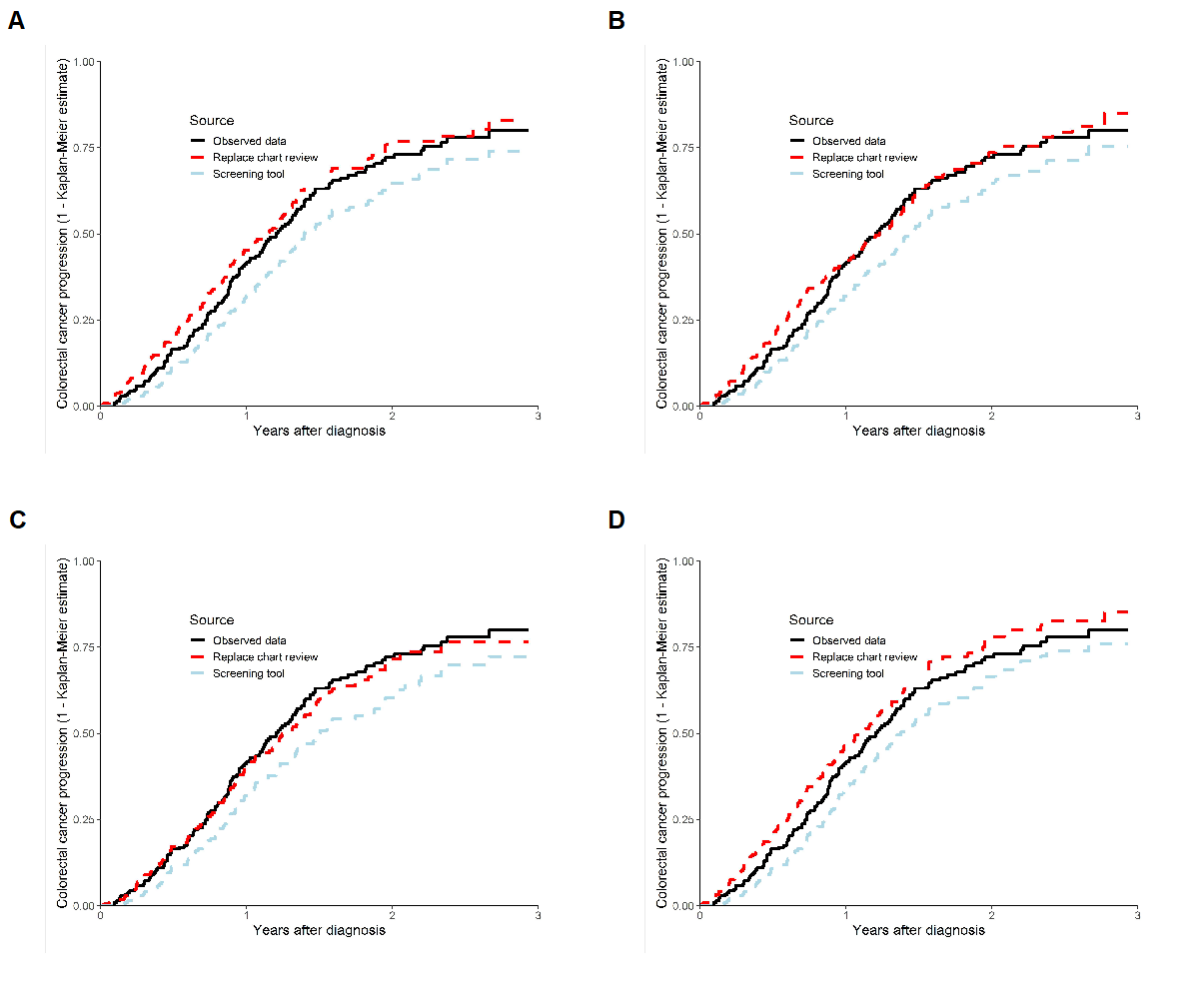
Kaplan-Meier curves of time to progression for the observed data and through the use of Clinical-BigBird and Clinical-Longformer in the colorectal test dataset (A: 2-class Clinical-BigBird; B: 3-class Clinical-BigBird; C: 2-class Clinical-Longformer; and D: 3-class Clinical-Longformer).

The test dataset included 225 individuals, of whom 127 (56.4%) had at least 1 EHR indicating cancer progression. Because the larger token models indicated higher accuracy than the Bio+ClinicalBERT models, an additional output was generated to evaluate their performance. All 4 larger token models (Clinical-BigBird and Clinical-Longformer, each with 2- and 3-class outcomes) slightly underestimated progression rates as a screening tool (Figure 2), but provided good approximations of cancer progression rates when replacing a chart review. When the data generated were included in Cox regression models with age at diagnosis as a predictor, the Clinical-BigBird 3-class and Clinical-Longformer 2-class models provided results that were similar to the observed data when used as a screening tool (Table 5). Only the Clinical-BigBird 3-class model provided results similar to the observed data when used to replace a chart review.

**Table 5:** Cox regression estimates (age predictor: ≥ 65 years vs <65 years) for the colorectal cancer test cohort.

Model		HR^a^ (95% CI)
Observed		1.23 (0.87-1.75)
**Screening tool**		
	Clinical-BigBird 2-class	1.31 (0.89-1.92)
	Clinical-BigBird 3-class	1.22 (0.84-1.79)
	Clinical-Longformer 2-class	1.27 (0.86-1.88)
	Clinical-Longformer 3-class	1.30 (0.89-1.90)
**Replace chart review**		
	Clinical-BigBird 2-class	1.41 (0.99-1.99)
	Clinical-BigBird 3-class	1.27 (0.89-1.79)
	Clinical-Longformer 2-class	1.37 (0.95-1.97)
	Clinical-Longformer 3-class	1.33 (0.95-1.87)

^a^HR: hazard ratio.

### Application of Models to Different Cancer Sites

When models developed by analyzing colorectal cancer data were applied to the breast cancer test datasets, the results were slightly lower than those obtained from the models developed using breast cancer data (Table 6). For example, the scaled Brier scores ranged from 0.58 to 0.69 when a model developed using colorectal cancer data was applied, but were 0.66 to 0.79 when a model developed using breast cancer data was applied.

**Table 6:** Results by model and outcome class for the model applied to different cancer sites.

Model, test dataset, and metrics			Bio+ClinicalBERT														Clinical-BigBird						Clinical-Longformer			
			2-class							3-class							2-class			3-class			2-class			3-class
			Head		Tail		Head and tail		Head			Tail		Head and tail												
**Colorectal cancer model applied to breast cancer test dataset**																										
	Sensitivity, %	76.3		76.8		76.3		76.1			75.9		73.0		*84.4* ^a^			83.8			77.1			82.0		
	PPV^b^, %	83.9		85.1		81.6		78.3			*87.0*		83.3		81.4			83.3			*87.0*			82.7		
	AUC^c^	0.96		0.96		0.94		0.95			0.95		0.95		*0.97*			*0.97*			0.95			0.96		
	Scaled Brier score	0.62		0.63		0.61		0.58			0.65		0.60		0.65			0.68			*0.69*			0.66		
	Charts reduced, %	88.2		88.3		87.9		87.4			*88.7*		88.6		86.6			86.9			88.5			87.1		
**Breast cancer model applied to colorectal cancer test dataset**																										
	Sensitivity, %	69.4		63.4		75.4		70.7			63.0		70.3		68.5			83.6			*86.8*			82.4		
	PPV, %	80.6		82.5		76.3		80.9			82.9		77.7		*86.8*			74.6			70.8			74.0		
	AUC	0.97		0.96		0.97		0.97			0.96		0.97		0.97			*0.98*			*0.98*			*0.98*		
	Scaled Brier score	0.55		0.52		0.55		0.57			0.55		0.55		0.58			*0.61*			0.55			*0.61*		
	Charts reduced, %	90.8		91.8		89.5		90.7			*91.9*		90.4		91.2			88.1			86.9			88.1		

^a^Italicization indicates the highest value.

^b^PPV: positive predictive value.

^c^AUC: area under the curve.

When models developed by analyzing breast cancer data were applied to the colorectal cancer test datasets, the results were also slightly lower than those obtained from the models developed using colorectal cancer data (Table 6). For example, the scaled Brier scores ranged from 0.52 to 0.61 when a model developed using breast cancer data was applied but were 0.49 to 0.65 when a model developed using colorectal cancer data was applied.

### Identifying Influential Words in EHRs

Using the Clinical-Longformer 2-class model, influential words were identified by modifying EHRs and reporting differences in predicted probabilities (Multimedia Appendix 1). In addition, all EHRs sampled were provided with ID values for reference (B_1-B_6 for breast cancer and C_1-C_6 for colorectal cancer). The most common influential word identified from the sample EHRs was *progression*. In both the breast and colorectal models, the presence of the word *progression* early in the chart had a larger impact. For example, the predicted probability of sample B_2 decreased from >0.99 to <0.01 when the word *progression* was removed early in the chart. The predicted probability of sample C_2 decreased from 0.99 to 0.08 when the first 2 occurrences of *progression* were removed, despite having 4 additional occurrences of *progression* later in the EHR. The effect of adding the word *progression* to EHRs was inconsistent. When it was added to the first sentence of samples B_1, B_3, B_5, B_6, C_1, C_3, C_5, and C_6, the predicted probabilities increased to 0.70, 0.60, 0.99, 0.98, 0.86, 0.59, 0.88, and 0.57, respectively.

Predicted probabilities were also influenced by the frequency of tokens within an EHR. Adding *progression* twice to EHRs that did not mention *progression* had an inconsistent impact. Although *progression* was added twice in B_3 and C_1, the predicted probabilities were only 0.21 and <0.01, respectively. In contrast, adding *progression* twice to B_1 and C_3 increased predicted probabilities to 0.79 and 0.96, respectively. Brain metastases were mentioned twice in a false-negative sample (B_5). By removing the first occurrence of *brain*, the predicted probability decreased from 0.49 to 0.04. When *brain* was added to the first sentence, the predicted probability increased to 0.63.

Word combinations were also influential. The expression *disease is progressing* increased the predicted probabilities more than adding only the words *disease* or *progression* separately. For example, when this expression was added to the first sentences of samples B_1, B_3, and C_1, the predicted probabilities were 0.91, 0.92, and 0.98 versus 0.70, 0.60, and 0.86 when only *progression* was added. However, the predicted probability remained 0.59 for sample C_3 when either the term *progression* or the phrase *disease is progressing* was added to the first sentence. Despite the inclusion of *progression*, the addition of *no evidence of progression* and *has not progressed yet* was associated with limited increases in predicted probabilities for true-negative and false-negative EHRs (values ranging <0.01-0.22 for samples B_1, B_3, C_1, and C_3).

Although none of the sample EHRs contained the words *relapse* or *recurrence*, these terms were sometimes used interchangeably with cancer progression (ie, tumor growth occurring without remission) in the physician notes reviewed. When *relapse* was added to the first sentence of true-negative and false-negative samples, the effect on predicted probabilities was inconsistent: 0.07 for B_1, 0.94 for B_3, 0.33 for C_1, and <0.01 for C_3. When *recurrence* was added to the first sentence of true-negative and false-negative samples, the effect on predicted probabilities was also inconsistent: 0.17 for B_1, 0.89 for B_3, 0.91 for C_1, and 0.39 for C_3.

Similarly, the false-negative B_3 sample described an individual with breast cancer who had worsened disease and was subsequently enrolled in the palliative care program (predicted probability of 0.04). The false-negative C_3 sample described an individual with colorectal cancer who had a substantial increase in carcinoembryonic antigen test values that required a change in chemotherapy regimen (predicted probability of <0.01). Although these EHRs included text describing cancer progression, the predicted probabilities generated were near 0. These terms may have been too infrequent in the training data for the language models to recognize them.

## Discussion

### Principal Findings

The larger token models (Clinical-BigBird and Clinical-Longformer, which can include up to 4096 tokens) demonstrated higher accuracy in capturing cancer progression in EHRs than the Bio+ClinicalBERT models (which can include up to 512 tokens). All larger token models trained on breast cancer data provided good approximations of the observed data when used as a screening tool (ie, provided good approximations for both KM estimates and HRs from a Cox regression model) and could remove approximately 85% of EHRs from the chart review process. The Clinical-BigBird 2-class model also provided good approximations for the observed data when used to replace a chart review. In contrast, the only model trained on colorectal cancer data that provided good approximations for both KM estimates and HRs from a Cox regression model was the Clinical-BigBird 3-class model to replace a chart review.

The models that were generated were not strongly influenced by cancer-site–specific terms (eg, site of cancer progression, site for radiation therapy, and chemotherapy regimen). Breast cancer models applied to colorectal cancer data were only slightly less accurate than colorectal cancer models, and colorectal models applied to breast cancer data were only slightly less accurate than breast cancer models. In addition, cancer-specific terms were not influential when removing or adding words from sample EHRs and generating predicted probabilities. The results from identifying influential words in EHRs suggest that the appearance of the word *progression* earlier in a chart led to higher accuracy compared to when it appeared later in a chart. This could explain why the Bio+ClinicalBERT model that analyzed the tail of EHRs had the lowest scaled Brier scores among the models. This may be because cancer progression more commonly appeared at the beginning of EHRs and allowed better pattern recognition for models that included this information. The lack of cancer-site–specific information could increase the applicability of the current models to a variety of cancer sites. However, larger datasets may be required to identify tokens missed by the current models that would be needed to distinguish the use of *progression* to describe cancer progression (eg, tumor growth and associated change in treatment) from other uses of progression (eg, a patient is progressing well in their treatment), such as terms indicating diagnostic uncertainty and diagnostic procedures. In this situation, the 3-class model would be advantageous in distinguishing between these 2 classes of terms. The results also indicated that models included relationships between tokens where sometimes combinations of words could increase accuracy more than individual words (eg, *disease is progressing* vs only *progression* or *no evidence of progression*). Sometimes the words *relapse* and *recurrence* were used to describe cancer progression (ie, tumor growth without remission), and their less frequent use may explain why they were less influential than the more commonly used word *progression*. In addition, the inconsistent impact of tokens could be due to the absence or presence of other tokens found in an EHR.

Although larger token models demonstrated higher accuracy relative to the Bio+ClinicalBERT models, a limited number of tokens appeared to be strongly captured by the models evaluated for influential tokens. If limited tokens are required to identify cancer progression, providing outcome labels for individual sentences rather than individual EHRs could increase accuracy by limiting the number of tokens that need to be considered, which would facilitate a model’s ability to recognize patterns in the analyzed text. In addition, analyzing data at the sentence level would remove the impact of token location within EHRs. However, if limited tokens related to cancer progression are found to be influential because of inadequate examples of less commonly used terminology, larger datasets may be required.

All models demonstrated near-perfect AUC scores (ie, 0.95-0.99). However, AUC only reports whether predicted probabilities are higher for cases than for controls [8,9]. This ignores how close predictions are to outcome labels, as well as the rate of the outcome [10]. Due to these limitations, AUC lacks sensitivity when comparing different models [8]. In contrast, the scaled Brier score is a metric that accounts for the difference between predicted probabilities and outcome labels, while also accounting for the outcome rate [11]. This increases the sensitivity to detect differences between the models. For example, AUC values ranged from 0.95 to 0.99, but scaled Brier scores ranged from 0.49 to 0.79. Although a scaled Brier score of 0.79 may indicate near-perfect prediction, it does not indicate the impact of error (eg, the level of overestimation or underestimation). Because the purpose of the models is to simplify the chart review process, the ideal evaluation would be to compare the data generated from a model with the data generated from a chart review (eg, KM curves and Cox regression estimates compared with the observed data).

### Limitations

Comparisons between generated data and chart review data could be biased (ie, KM curves and Cox regression HRs). The test dataset for breast cancer included 77 individuals with a cancer progression event, and the test dataset for colorectal cancer included 127 individuals with a cancer progression event. It has been suggested that external datasets should have at least 100 or 200 events [12-14] (and potentially more [15]) to effectively evaluate model performance. Although the other output reported (eg, sensitivity and PPV) was based on more than 200 events of cancer progression, this output may still be misleading. The previously mentioned recommendations were based on the performance of statistical models, which require substantially less data to achieve the same performance as machine learning models [16] or the same variability in performance [17].

Results generated for the test datasets substantially improved for some models, were relatively unchanged, or were worse in comparison with the validation dataset. Some of the improvement can be expected from generating models that analyze data by combining the training and validation datasets (ie, increased sample size). However, different results could have been obtained from selecting different seeds. Reporting the average and variation of performance metrics across models with different seeds (eg, dozens) would provide more reliable evaluations. This has previously been demonstrated in statistical models when comparing results from split-sampling to bootstrapping [18,19], where split-sampling (ie, reporting the performance of 1 model) required substantially more data than bootstrapping (ie, average across multiple models) to achieve the same level of accuracy. However, the feasibility of averaging model performance metrics depends on computational resources, model complexity, and dataset sizes. For example, the processing time for the training datasets of Clinical-BigBird or Clinical-Longformer models in this study was more than 6 hours using a workstation with 2 NVIDIA RTX A5000 GPUs with 24 GB RAM each. Calculated floating-point operations per second (FLOPS) in single-precision (FP32) operations varied from approximately 750 GigaFLOPS with 2-class Bio+ClinicalBERT models to 13 TeraFLOPS with 3-class larger token models. A larger dataset would be required to generate more accurate models, which would also increase the processing time.

The cancer cohort consisted of Manitoba residents and only included breast and colorectal cancer cases. The results may not be generalizable to other geographic locations where clinical notes and terminology may differ. Model generalizability would need to be evaluated with additional cancer sites.

### Conclusions

In conclusion, the Clinical-BigBird and Clinical-Longformer models were able to generate tools to aid in screening EHRs from Manitoba, Canada, to identify breast and colorectal cancer progression. They could potentially remove at least 84% of EHRs from the chart review process to generate data for future retrospective research studies using breast and colorectal cancer EHRs. The colorectal models demonstrated more errors than the breast cancer models. In addition, the models appeared to include limited tokens to generate predictions. Additional research may be required to improve model performance. For example, improvements could be obtained by increasing the training dataset size and by analyzing EHRs at the sentence level rather than at the chart level. Both factors could increase the number of relevant tokens captured by the models for identifying cancer progression.

## Appendix

Multimedia Appendix 1 Results of the addition and removal of words and partial sentences to identify influential words.

## Abbreviations

AUC: area under the curve
BERT: bidirectional encoder representations from transformers
EHR: electronic health record
FLOPS: floating-point operations per second
HR: hazard ratio
KM: Kaplan-Meier
PPV: positive predictive value
