# Supplementary material for: Deep Learning Models to Screen Electronic Health Records for Breast and Colorectal Cancer Progression: Performance Evaluation Study
Source: JMIR AI. 2025 Oct 13;4:e63767. doi: 10.2196/63767 (PMC12559821; doi:10.2196/63767)
Supplement: Multimedia Appendix 1 [file ai_v4i1e63767_app1.pdf]

Table S1. Results of addition and removal of words and partial sentences to identify influential words

Cancer site	Classification	ID	Number of words in EHR	Changes	Probability
Breast	True negative	B_1	95	None	<0.01
				Add "progression" in middle of chart	0.69
				Add "progression" in middle and end of chart	0.79
				Add "progression" in first sentence	0.70
				Add "relapse" in first sentence	0.07
				Add "recurrence" in first sentence	0.17
				Add "disease" in first sentence	<0.01
				Add "disease is progressing" in first sentence	0.91
				Add "no evidence of progression" in first sentence	0.00
				Add "has not progressed yet" in first sentence	0.08
	True positive	B_2	828	None	>0.99
				Remove "progression" early in chart	<0.01
	False negative	B_3	188	None	0.04
				Add "progression" in middle of chart	0.24
				Add "progression" in middle and end of chart	0.21
				Add "progression" in first sentence	0.60
				Add "relapse" in first sentence	0.94
				Add "recurrence" in first sentence	0.89
				Add "disease" in first sentence	0.05
				Add "disease is progressing" in first sentence	0.92
				Add "no evidence of progression" in first sentence	0.22
				Add "has not progressed yet" in first sentence	0.09
	False positive	B_4	593	None	>0.99
				Remove "progressing" x1 in first half of chart	>0.99
				Remove "progressing" x2 in first half of chart	0.82
				Remove "progressing" x2 (in first half of chart) and "progression" x1 (in end of chart)	0.82
				Remove "disease" x3	>0.99
				Remove all chemotherapy regimens	>0.99
				Remove all "progressing", "progression", and "disease"	0.16
				Remove all "progressing", "progression", and chemotherapy regimens	0.50

				Remove all "progressing", "progression", "disease", and chemotherapy regimens	0.02
	False negative	B_5	219	None	0.49
				Remove "developed brain metastases" in middle of chart	<0.01
				Remove "brain" x1 in middle of chart	0.04
				Remove "brain" x2 in middle and end of chart	<0.01
				Add "progression" in first sentence	0.99
				Add "brain" in first sentence	0.63
	False positive	B_6	963	None	0.50
				Remove "progression" in first half of chart	0.09
				Add "progression" in first sentence	0.98
Colorectal	True negative	C_1	160	None	<0.01
				Add "progression" in middle of chart	<0.01
				Add "progression" in middle and end of chart	<0.01
				Add "progression" in first sentence	0.86
				Add "relapse" in first sentence	0.33
				Add "recurrence" in first sentence	0.91
				Add "disease" in first sentence	<0.01
				Add "disease is progressing" in first sentence	0.98
				Add "no evidence of progression" in first sentence	0.00
				Add "no evidence of progression" in first sentence	0.00
	True positive	C_2	840	None	0.99
				Remove "progression" x1 early in the chart	0.99
				Remove "progression" x2 early in the chart	0.08
				Remove "progression" x6	<0.01
	False negative	C_3	211	None	<0.01
				Add "progression" in middle of chart	0.59
				Add "progression" in middle and end of chart	0.96
				Add "progression" in first sentence	0.59
				Add "relapse" in first sentence	<0.01
				Add "recurrence" in first sentence	0.39
				Add "disease" in first sentence	<0.01
				Add "disease is progressing" in first sentence	0.59
				Add "no evidence of progression" in first sentence	0.00
				Add "has not progressed yet" in first sentence	0.00

False positive	C_4	105	None	0.99
			Remove "progression" in first half of chart	<0.01
False negative	C_5	400	None	0.50
			Remove "progression" early in chart	<0.01
			Add "progression" in middle of chart	0.50
			Add "progression" in first sentence	0.88
False positive	C_6	257	None	0.50
			Remove "progression" in first half of chart	<0.01
			Add "progression" in end of chart	0.51
			Add "progression" in first sentence	0.57
